# Interep: An R Package for High-Dimensional Interaction Analysis of the Repeated Measurement Data

**DOI:** 10.3390/genes13030544

**Published:** 2022-03-19

**Authors:** Fei Zhou, Jie Ren, Yuwen Liu, Xiaoxi Li, Weiqun Wang, Cen Wu

**Affiliations:** 1 Department of Statistics, Kansas State University, Manhattan, KS 66506, USA; feiz@ksu.edu (F.Z.); yuwen@ksu.edu (Y.L.); xiaoxili@ksu.edu (X.L.); 2 Department of Biostatistics and Health Data Sciences, Indiana University School of Medicine, Indianapolis, IN 46202, USA; jr117@iu.edu; 3 Department of Food, Nutrition, Dietetics and Health, Kansas State University, Manhattan, KS 66506, USA; wwang@ksu.edu

**Keywords:** GEE, interaction analysis, longitudinal data, penalized variable selection

## Abstract

We introduce *interep*, an R package for interaction analysis of repeated measurement data with high-dimensional main and interaction effects. In G × E interaction studies, the forms of environmental factors play a critical role in determining how structured sparsity should be imposed in the high-dimensional scenario to identify important effects. Zhou et al. (2019) (PMID: 31816972) proposed a longitudinal penalization method to select main and interaction effects corresponding to the individual and group structure, respectively, which requires a mixture of individual and group level penalties. The R package *interep* implements generalized estimating equation (GEE)-based penalization methods with this sparsity assumption. Moreover, alternative methods have also been implemented in the package. These alternative methods merely select effects on an individual level and ignore the group-level interaction structure. In this software article, we first introduce the statistical methodology corresponding to the penalized GEE methods implemented in the package. Next, we present the usage of the core and supporting functions, which is followed by a simulation example with R codes and annotations. The R package *interep* is available at The Comprehensive R Archive Network (CRAN).

## 1. Introduction

In longitudinal studies, the same subjects are repeatedly measured over a set of units, such as a period of time. The correlated nature of the repeated measurements has motivated the development of a wide spectrum of approaches that account for intra-cluster interconnections [1,2]. One of the representative examples is the generalized estimating equation (GEE), originally proposed by Liang and Zeger [3]. It only depends on the first two marginal moments of repeated measurements and a working correlation matrix to fully incorporate the intra-cluster correlations of longitudinal data. Such a feature is particularly appealing when the full likelihood function is difficult to specify under non-Gaussian assumptions. In the past decade, variable selection has gradually become one of the central tasks in longitudinal studies due to the explosion of the use of high-dimensional data. In the literature, Wang et al. [4] developed the penalized generalized estimating equation (PGEE) with LASSO [5] to select important features. Although GEE and PGEE are robust when the working correlations are misspecified, the resulting estimators can be inefficient. To overcome this limitation, Cho and Qu [6] developed the penalized quadratic inference function (PQIF) method for variable selection with penalty functions such as SCAD [7], LASSO and adaptive LASSO [8], respectively.

For repeated-measurement data, regularized estimation with the above penalty functions continuously shrinks the regression coefficients towards zero. If the tuning parameters are properly chosen, regression coefficients corresponding to unimportant features will be estimated as zero, whereas those for important ones are still nonzero. Therefore, variable selection and parameter estimation can be achieved at the same time in the longitudinal setting. Despite the success, these penalization methods, including those of Wang et al. [4] and Cho and Qu [6], among many others, mainly focus on selecting the main effects and ignore the interactions. Variable selection methods that do not appropriately account for the interaction structure generally yield inaccurate estimation results and false findings [9]. Use the gene–environment (G × E) interaction as an example. The G × E interactions are typically modeled as the product terms between low-dimensional E factors and high-dimensional G factors. The identification of important genetic main effects and G × E interactions are of primary interest since the E factors are usually determined based on existing studies and are not subject to selection. This essentially requires sparse group or bi-level selection [10,11,12]. With the repeated measure response, although PGEE and PQIF can still be adopted for G × E interactions by treating interactions as the main effects, they no longer lead to the optimal estimation and identification results [13,14].

The environmental factors take a very unique form in the longitudinal G × E study conducted by Zhou et al. [13]. The interaction effects were initially modeled as the product of lipidomics features and a treatment factor consisting of four different levels. Since the E factor is then represented as three dummy variables (or binary indicators), the identification of important lipids by environmental interactions requires the selection to be performed in a “group–in, group–out” manner, as in the group LASSO [15]. Such a formulation eventually leads to a mixture of individual- and group-level penalty functions used to identify genetic main effects and G × E interactions simultaneously under the longitudinal outcome which was the bodyweight of CD1 mice, repeatedly measured over 10 consecutive weeks.Please refer to Zhou et al. [13] and King et al. [16] for more detailed descriptions of the data. Note that although Zhou et al. [13] was partially driven by an animal model, the proposed methods are potentially applicable in G × E studies of human diseases as long as the selection of interactions is conducted in a group manner.

In this article, we demonstrate the R package *interep* [17] that implements the GEE-based penalization methods proposed in Zhou et al. [13] for the simultaneous selection of main and interaction effects on the individual and group level, correspondingly. These procedures assume linear G × E interactions [9], which have not been examined in high-dimensional longitudinal studies to date. On the other hand, the nonlinear G × E interactions [18,19,20] are usually dissected through the varying coefficient model and its variants, which can be analyzed using the high-dimensional longitudinal varying coefficient models in studies such as [21,22].

The open-source R package *interep* is publicly available on CRAN at https://cran.r-project.org/package=interep (accessed on 11 March 2022) [17]. To facilitate rapid computation, we developed the core modules of the R package in C++. The rest of the paper is organized as follows. In Section 2, we provide an overview of penalized interaction analysis in longitudinal studies. Section 3 describes the main and supporting functions implemented in the *interep* package. Simulation examples designed to showcase the usage of the package are presented in Section 4. We conclude the paper with discussions in Section 5.

## 2. Statistical Methods

### 2.1. Data Structure and Model Setup for the Longitudinal Interaction Analysis

In the longitudinal G × E study with *n* subjects, we observe $t_{i}$ repeated measurements for the ith (1⩽i⩽n) subject over time. $Y_{ij}$ represents the response (or phenotype) for the ith subject at time *j* ($1⩽j⩽t_{i}$). $G_{ij}={\left(G_{ij1},\ldots ,G_{ijp}\right)}^{⊤}$ denotes the *p*-dimensional vector of genetic factors. $E_{ij}={\left(E_{ij1},\ldots ,E_{ijq}\right)}^{⊤}$ is the *q*-dimensional environmental factors coded for the treatment with (*q* + 1) levels. Henceforth, we assume $t_{i}=t<\infty$ without the loss of generality. The G × E model consists of the genetic and environmental main effects, and the G × E interactions, which are associated with the repeatedly measured phenotypic trait, can be specified as follows:

(1) $$Y_{ij}=\beta_{n0}+E_{ij}^{⊤}\beta_{n1}+G_{ij}^{⊤}\beta_{n2}+{\left(G_{ij}⊗E_{ij}\right)}^{⊤}\beta_{n3}+\epsilon_{ij}=Z_{ij}^{⊤}\beta_{n}+\epsilon_{ij},$$

where $\beta_{n}={\left(\beta_{n0},\beta_{n1}^{⊤},\beta_{n2}^{⊤},\beta_{n3}^{⊤}\right)}^{⊤}$ and $Z_{ij}={\left(1,E_{ij}^{⊤},G_{ij}^{⊤},{\left(G_{ij}⊗E_{ij}\right)}^{⊤}\right)}^{⊤}$ are (pq+p+q+1)-dimensional vectors denoting all the main and interaction effects. $\beta_{n0}$ is the intercept. $\beta_{n1}$, $\beta_{n2}$, and $\beta_{n3}$ are unknown coefficient vectors of dimensions *q*, *p*, and pq, respectively. Furthermore, $G_{ij}⊗E_{ij}$ is the pq-dimensional vector of the Kronecker product in the following form:

$$G_{ij}⊗E_{ij}={\left[G_{ij1}E_{ij1},G_{ij1}E_{ij2},\ldots ,G_{ij1}E_{ijq},G_{ij2}E_{ij1},\ldots ,G_{ijp}E_{ijq}\right]}^{⊤},$$

which is adopted to model the G × E interactions. The observations measured from the same subjects are correlated, and are assumed to be independent if they are from different subjects. The random error $\epsilon_{ij}$ (1⩽j⩽t) has a mean zero and a finite variance. For convenience, we assume that $\epsilon_{i}={\left(\epsilon_{i1},\ldots ,\epsilon_{it}\right)}^{T}$ follows a multivariate normal distribution $N_{t}\left(0,\Sigma_{i}\right)$, with $\Sigma_{i}$ as the covariance matrix for the repeated measure of the ith subject across the *t* time points.

### 2.2. An Overview of Generalized Estimating Equations in the Interaction Analysis

In the literature, G × E interactions with multiple phenotypes have been shown to be effective when the correlations among multivariate measurements can be efficiently accommodated [23]. The intra-correlative nature of longitudinal data makes it difficult to specify the joint likelihood function, especially when the responses are not normal. Liang and Zeger [3] developed the generalized estimating equation (GEE) framework to incorporate the correlations of repeated measurements from the same subject. GEE conducts marginal regression using a quasi-likelihood function. It relaxes the distributional assumption by only requiring the specification of the first two moments and a working correlation matrix for $Y_{ij}$. We express the first two marginal moments of $Y_{ij}$ as $E\left(Y_{ij}\right)=\mu_{ij}=Z_{ij}^{T}\beta_{n}$ and $\mathrm{Var}\left(Y_{ij}\right)=\upsilon \left(\mu_{ij}\right)$, correspondingly. Here, υ denotes a known variance function. The estimating equation for $\beta_{n}$ can then be written as:

(2) $$\sum_{i=1}^{n}\frac{\partial \mu_{i}\left(\beta_{n}\right)}{\partial \beta_{n}}V_{i}^{-1}\left(Y_{i}-\mu_{i}\left(\beta_{n}\right)\right)=0,$$

where $\mu_{i}\left(\beta_{n}\right)={\left(\mu_{i1}\left(\beta_{n}\right),\ldots ,\mu_{it}\left(\beta_{n}\right)\right)}^{⊤}$ and $Y_{i}={\left(Y_{i1},\ldots ,Y_{it}\right)}^{⊤}$. $V_{i}$ is the covariance matrix of $Y_{i}$. In Equation (2), $\frac{\partial \mu_{i}\left(\beta_{n}\right)}{\partial \beta_{n}}$ is equivalent to $Z_{i}={\left(Z_{i1},\ldots ,Z_{it}\right)}^{⊤}$ for the t×(pq+p+q+1) matrix of the genetic main effects and G × E interactions.

Liang and Zeger [3] proposed the estimation of $V_{i}$, the t×t covariance matrix for the *i*th subject, through a working correlation matrix R(η) as $V_{i}=A_{i}^{\frac{1}{2}}\left(\beta_{n}\right)R\left(\eta \right)A_{i}^{\frac{1}{2}}\left(\beta_{n}\right)$, where the diagonal marginal variance matrix is defined as $A_{i}\left(\beta_{n}\right)\;=\;\mathrm{diag}\left\{\mathrm{Var}\left(Y_{i1}\right),\ldots ,\mathrm{Var}\left(Y_{it}\right)\right\}$, and the t×t working correlation matrix R(η) is indexed by a finite-dimensional vector of nuisance parameters η. Widely chosen correlation structures for R(η) include first-order auto-regressive or AR(1), exchangeable and independent structures, among others. One of the defining features of GEE, as shown by Liang and Zeger [3], is that the GEE estimator of regression coefficients can be consistently estimated if η can be consistently estimated, and that this consistency holds even when the working correlation is misspecified.

### 2.3. Penalized Identification of G × E Interactions in the Longitudinal Study

In this study, the environmental factors of interest denote a group of binary indicators representing treatment levels. Although this group’s dimensionality is limited, given the high-dimensional genetic factors and the G × E interactions, the identification of important effects requires variable selection. Representative longitudinal variable selection methods, including PGEE [4] and PQIF [6], aim at selecting the main effects and ignore the interactions. Furthermore, the G × E interaction modeled in Equation  (1) is between the G factor and all the *q* factor levels. Its selection is in a “group in, group out” spirit, whereas the selection of main genetic factors is on the individual level. Thus, the tailored variable selection method can conduct shrinkage estimations on a mixture of group and individual levels, as proposed in Zhou et al. [13]. We first define U($\beta_{n}$) as the score equation for $\beta_{n}$:

$$U\left(\beta_{n}\right)=\sum_{i=1}^{n}Z_{i}^{T}V_{i}^{-1}\left(Y_{i}-\mu_{i}\left(\beta_{n}\right)\right).$$

To perform regularized selection, we adopt the minimax concave penalty (MCP) as the baseline penalty function as follows, due to its computational convenience and rigorous theoretical properties [24]:

$$\rho \left(m;\lambda ,\gamma \right)=\lambda \int_{0}^{\left|m\right|}{\left(1-\frac{x}{\gamma \lambda }\right)}_{+}dx,$$

where λ is the tuning parameter and γ is the regularization parameter, with the first derivative:

$$\rho \prime \left(m;\lambda ,\gamma \right)=\left(\lambda -\frac{m}{\gamma }\right)I\left(|m|\leq \gamma \lambda \right).$$

Then, the penalized generalized estimating equation for the interaction analysis of repeated measure data is:

(3) $$Q\left(\beta_{n}\right)=U\left(\beta_{n}\right)-\sum_{g=1}^{p}\rho \prime (|\beta_{n2g}|;\lambda_{1},\gamma )\mathrm{sign}\left(\beta_{n2g}\right)-\sum_{h=1}^{p}\rho \prime (||\beta_{n3h}{\left|\right|}_{\Sigma_{h}};\sqrt{q}\lambda_{2},\gamma ),$$

where coefficient $\beta_{n2g}$ corresponds to the *g*th genetic main effect, and coefficient vector $\beta_{n3h}$ represents the effect of interaction between the *h*th G factor and E factors. The penalty term involving $\beta_{n2g}$ imposes individual-level shrinkage to select G factors. Meanwhile, since the E factors are in the form of a group which should be chosen or discarded in a group-wise manner, the penalty term with respect to $\left|\right|\beta_{n3h}{\left|\right|}_{\Sigma_{h}}$ conducts group-level regularization to select G × E interactions through penalizing the empirical norm $\left|\right|\beta_{n3h}{\left|\right|}_{\Sigma_{h}}$. Here, $\left|\right|\beta_{n3h}{\left|\right|}_{\Sigma_{h}}={\left(\beta {’}_{n3h}\Sigma_{h}\beta_{n3h}\right)}^{1/2}$ with $\Sigma_{h}=n^{-1}B_{h}^{⊤}B_{h}$, where $B_{h}$ is the subset of the design matrix corresponding to the interaction between the E factor and the *h*th genetic factor. In addition, $\lambda_{1}$ and $\lambda_{2}$ in Equation (3) are tuning parameters controlling the amount of shrinkage in the selection of main and interaction effects.

Here, we adopt MCP as the baseline penalty due to its computational efficiency and accuracy, as observed in our past studies. Other popular choices include LASSO, adaptive LASSO and SCAD [25]. Although we conjecture that the alternative choices of baseline penalty function can be equally applicable, it is beyond the scope of our study to examine their pros and cons in the interaction studies. These baseline penalty functions and their extensions have been implemented in a number of R packages. For example, LASSO and relevant regularization methods (including ridge regression and elastic net) have been implemented in the *glmnet* package for generalized linear models [26]. In repeated-measurement studies, the R package *PGEE* has been developed for PGEE with the non-convex SCAD penalty [27]. Furthermore, please refer to R package https://CRAN.R-project.org/package=regnet (accessed on 11 March 2022) for estimation and variable selection in generalized linear models using MCP and its extensions [28,29]. In general, advanced regularization methods can be developed based on the baseline penalty functions to accommodate different patterns of sparsity [30,31,32]. In studies of complex diseases, bi-level structures are commonly found and thus motivate the development of statistical methods that efficiently incorporate such a hierarchy [33,34,35]. Regularization has been shown to be particularly efficient in accommodating bi-level or sparse group sparsity in G × E studies [9].

The dataset generated from a profiling study assessing lipid changes in weight-controlled mice [16] motivated the development of penalized longitudinal interaction methods in [13]. This dataset has the following characteristics. First, the genetic factor is defined as lipidomics features which have been rarely examined in a high-dimensional scenario using variable selection. Second, the lipid–environment interactions have a group structure, so the selection of main and interactive effects is supposed to be accommodated simultaneously on both the group and individual levels.

Nevertheless, the uniqueness of the dataset does not limit the scope of the proposed methods. The G factors can be any multi-omics measurements, such as mRNA expressions, single-nucleotide polymorphisms (SNPs) and copy number variations (CNVs), as long as their dimensionality is large. In terms of the special form of the environmental factors, although it was constructed based on a treatment with exercise and/or dietary restrictions with multiple factor levels, the proposed methods are still applicable if the structure of E factors results in group-level selection. For instance, in cancer genomics studies, the stage of cancer is a categorical variable usually with more than two levels, which can lead to the group-level interaction structure.

### 2.4. Computational Algorithms

We implemented the Newton–Raphson algorithm in the *interep* [17] package for the efficient estimation of $\hat{\beta }$ under the objective function (3). The algorithm proceeds iteratively. At the (d+1)th iteration,

(4) $${\hat{\beta }}_{n}^{d+1}={\hat{\beta }}_{n}^{d}+{\left[T\left({\hat{\beta }}_{n}^{d}\right)+nW\left({\hat{\beta }}_{n}^{d}\right)\right]}^{-1}\left[U\left({\hat{\beta }}_{n}^{d}\right)-nW\left({\hat{\beta }}_{n}^{d}\right){\hat{\beta }}_{n}^{d}\right],$$

where $U({\hat{\beta }}_{n}^{d})$ is the score function in ${\hat{\beta }}_{n}^{d}$ and $T({\hat{\beta }}_{n}^{d})$ is defined as the first-order derivative function of $U({\hat{\beta }}^{d})$:

$$T\left({\hat{\beta }}_{n}^{d}\right)=\sum_{i=1}^{n}Z_{i}^{T}V_{i}^{-1}Z_{i}.$$

In the objective function (3), the MCP penalty is utilized on both the individual and group level to detect main and interactive effects. Accordingly, $W({\hat{\beta }}_{n}^{d})$ is a diagonal matrix with the diagonal entries corresponding to the first-order derivatives of MCP and group MCP as follows:

$$\begin{array}{c} W\left({\hat{\beta }}_{n}^{d}\right)=\mathrm{diag}\{\underset{1+q}{\underset{︸}{0,\ldots ,0}},\frac{\rho^{\prime }\left(\right|{\hat{\beta }}_{n21}^{d}\left|;\lambda_{1},\gamma \right)}{\epsilon +|{\hat{\beta }}_{n21}^{d}|},\ldots ,\frac{\rho^{\prime }\left(\right|{\hat{\beta }}_{n2p}^{d}\left|;\lambda_{1},\gamma \right)}{\epsilon +|{\hat{\beta }}_{n2p}^{d}|},\\ \frac{\rho^{\prime }\left(|\right|{\hat{\beta }}_{n31}^{d}{\left|\right|}_{\Sigma_{1}};\sqrt{q}\lambda_{2},\gamma )}{\epsilon +\left|\right|{\hat{\beta }}_{n31}^{d}{\left|\right|}_{\Sigma_{1}}},\ldots ,\frac{\rho^{\prime }\left(|\right|{\hat{\beta }}_{n3p}^{d}{\left|\right|}_{\Sigma_{p}};\sqrt{q}\lambda_{2},\gamma )}{\epsilon +\left|\right|{\hat{\beta }}_{n3p}^{d}{\left|\right|}_{\Sigma_{p}}}\}, \end{array}$$

where ϵ is a small positive fraction (e.g., $10^{-6}$) to ensure numerical stability when the denominator is close to zero. The first (*q* + 1) elements on the diagonal of $W({\hat{\beta }}^{d})$ are zero, indicating that the intercept and environmental factors are not under regularized selection. The terms $nW\hat{\beta_{n}}$ and nW can be adopted to approximate the first-order derivative function of MCP in the penalized score equation and the second derivative function of the MCP penalty, respectively. With fixed tunings, the vector of regression parameters ${\hat{\beta }}_{n}^{d+1}$ is estimated iteratively until convergence is achieved. The update of ${\hat{\beta }}_{n}^{d+1}$ is terminated if the magnitude of the L1 difference between ${\hat{\beta }}_{n}^{d}$ and ${\hat{\beta }}_{n}^{d+1}$ is below a cutoff (e.g., $10^{-3}$). We observe that the convergence is usually achieved within 10 iterations.

The penalized GEE (3) involves two tuning parameters $\lambda_{1}$ and $\lambda_{2}$, and a regularization parameter γ. $\lambda_{1}$ and $\lambda_{2}$ determine the level of sparsity in MCP and group MCP, separately. The regularization parameter γ balances the smoothness of the resulting estimator and its unbiasedness. The insensitivity of specifying γ has been observed by Zhou et al. [13] and other studies [28,29]. We adopt *K*-fold cross-validation to determine the optimal pair of ($\lambda_{1},\lambda_{2}$). The dataset is split into *K* non-overlapping, roughly equal-sized parts. The *k*th (*k* = 1, …, *K*) fold is held out as the testing data, and the rest are used as training data. We use $n_{-k}$ and $n_{k}$ as the index sets to denote subjects in the testing and training set, respectively. Then the prediction error can be computed as

$${\mathrm{Pred}}_{-k}\left(\lambda_{1},\lambda_{2}\right)=\frac{1}{|n_{-k}|}\sum_{i\in n_{-k}}{\left(Y_{i}-\mu_{i}\left({\hat{\beta }}_{n_{k}}\right)\right)}^{2},$$

where ${\hat{\beta }}_{n_{k}}$ is the penalized estimate obtained based on the training data, and $|n_{-k}|$ is the cardinality of $n_{-k}$. The computation will be conducted for each one of the *K* folds, leading to a cross-validation error expressed as

(5) $$\mathrm{CV}\left(\lambda_{1},\lambda_{2}\right)=\frac{1}{K}\sum_{k=1}^{K}{\mathrm{Pred}}_{-k}\left(\lambda_{1},\lambda_{2}\right).$$

Over a two-dimensional grid of ($\lambda_{1},\lambda_{2}$), the best pair of tunings is chosen as the one yielding the smallest cross-validation error. The algorithm proceeds as follows:

1 Specify an appropriate range for the two-dimensional grid of ($\lambda_{1},\lambda_{2}$);
2 For a fixed ($\lambda_{1},\lambda_{2}$),
  (a) set an initial value to ${\hat{\beta }}_{n}^{0}$;
  (b) at the (d+1)th iteration, compute $T\left({\hat{\beta }}_{n}^{d}\right),W\left({\hat{\beta }}_{n}^{d}\right),U\left({\hat{\beta }}_{n}^{d}\right)$;
  (c) update ${\hat{\beta }}_{n}^{d+1}$ using Equation (4).
  (d) compute the cross-validation error based on Equation (5).
3 For each ($\lambda_{1},\lambda_{2}$) over the grid, repeat Step 2 until convergence and locate the optimal pair corresponding to the smallest cross-validation error.
4 Report ${\hat{\beta }}_{n}$ with respect to the optimal ($\lambda_{1},\lambda_{2}$).

Another popular strategy to pinpoint optimal tunings is based on independently-generated testing datasets. In simulation studies, as the data generating model is available, one can readily generate an independent testing dataset with a sample size much larger than the training data. In this way, the “cross” in CV is no longer necessary. The computation is thus less intensive and more efficient than cross-validation.

## 3. The R Package *Interep*

The *interep* package includes two main functions, interep and cv.interep. The function interep fits an GEE-based penalized interaction model to repeated-measure outcomes with high-dimensional main and interaction effects. The function cv.interep computes the cross-validation error over the two-dimensional grid of tuning parameters. In addition, there are supporting functions: dmcp, penalty and reformat. All these functions were developed by the authors. The core modules of the Newton–Raphson algorithm were written in C++. Therefore, the package is linked to Rcpp and RcppArmadillo [36,37,38].

### 3.1. The Main Functions

The standard R code for computing the regularized estimates with fixed tuning parameters provided by the *interep* package is

interep(e, g, y, beta0, corre, pmethod, lam1, lam2, maxits)

The function interep implements step 2 of the computational algorithms described in Section 2.4. The input argument e is a matrix of environment factors. In Zhou et al. [13], the environment factors form a group of dummy variables corresponding to the treatment. Such a form is not required to specify the input argument e here, which enables a wide usage of the package as long as selection on a group level is of interest. The input argument g is a matrix of G factors which can be lipidomics features in addition to popularly examined multi-omics measurements such as gene expressions, single-nucleotide polymorphisms (SNPs), DNA methylation and copy number variations (CNVs). Instead of directly specifying the entire design matrix as an input in R packages for regularized selection of the main effects, such as *glmnet* and *PGEE* [26,27], users of our package only need to provide the G and E factors, and the function interep will formulate the corresponding G × E design matrix accordingly. To offer users of the package more flexibility, we include beta0 as the argument for initial values. There are some typical choices for beta0 such as zeros or regularized estimates, using LASSO or ridge regression.

The character string argument corre can be used to specify the three working correlation structures implemented in Zhou et al. [13]. Specifically, corre = “i” denotes the independent correlation, whereas corre = “a” and corre = “e" denote AR-1 and exchangeable correlations, respectively. In addition to the proposed interaction analysis consisting of the selection of main effects on the individual level and interactions on the group level, Zhou et al. [13] includes an alternative method for comparison, which merely conducts selection on the individual level. The argument pmethod leads to the two major categories of methods under comparison. The proposed interaction method is called if corre = “mixed” is indicated, which demands an “MCP+ group MCP” type of penalty. On the other hand, corre = “individual” leads to the usage of the alternative methods, depending on MCP, to identify the effects.

The function interep computes regularized estimates for main effects and interactions under fixed tuning parameters which are provided by users as scalars for arguments lam1 and lam2, correspondingly. Note that when corre= “individual” is suggested, the second tuning parameter lam2 is not needed. The regularized estimation will only be conducted based on lam1. The input argument maxits is the maximum number of iterations allowed in the iterative algorithms.

In the *interep* package, the function cv.interep is closely related to the interep function described above. The R code is:

cv.interep (e, g, y, beta0, lambda1, lambda2, nfolds, corre,
pmethod, maxits)

The function cv.interep adopts the function interep to perform cross-validation over sequences of tuning parameters. Therefore, the common group of input arguments are shared between the two functions. One major difference lies in the arguments lambda1 and lambda2. These two arguments are no longer scalars. Instead, they are two user-supplied sequences for selecting individual-level main effects and group-level interaction effects, respectively. In the function cv.interep, we adopt the notations lambda1 and lambda2 to distinguish the sequences of tunings from the fixed ones (lam2 and lam2) used in the function interep. The cross-validation error will be computed over the two-dimensional grid of lambda1 and lambda2. The argument nfolds is the number of folds employed in cross validation.

### 3.2. Other Supporting Functions

In addition to the aforementioned core modules, the *interep* package also includes multiple supporting functions. MCP is the baseline penalty function adopted by Zhou et al. [13]; therefore, dmcp, the first-order derivative function of the MCP, is provided in the package. Selection can be conducted on the individual and group levels at the same time, or only on the individual level, which have been implemented through the function penalty. Argument pmethod = “mixed” refers to the bi-level selection. Similarly, pmethod = “individual” denotes the individual level selection, where the input for argument lam2 is no longer required. Furthermore, the function reformat changes the wide format of the repeated measurement to the long format. For example, with the phenotype repeatedly measured over five time points and a sample size of 250, the dimension of phenotype (or response) in wide format is 250 by 5. It changes to 1250 by 1 after applying reformat. Note that the matrix of main and interaction effects does not vary over time in Zhou et al. [13]. If the dimension is 250 by 75 for the argument x, then after reformatting, the dimension changes to 1250 by 76, including the intercept. Moreover, a simulated dataset, dat, is provided to demonstrate the penalized selection in the proposed longitudinal study. We provide more details in the next section.

## 4. Simulation

In this section, we will use a simulation example to demonstrate how to use the *interep* package to obtain regularized regression coefficients. The data-generating function is provided below.

Data1 <- **function** (n,p,k,**q**,rho){
# *n: sample size; p: number of G factors;*
# *k: number of time points; q: number of E factors*
y = **matrix** (**rep** (0,n*k),n,k)
sig = **matrix** (0,p,p)
**for** (i in 1: p) {
**for** (j in 1: p) { sig[i,j] = 0.5^**abs**(i-j)  }
}
x = mvrnorm(n,**rep**(0,p),sig)
g = x
 
# *generate binary variables*
dummy0 <- **as.numeric**(x[,1] <= -0.5)
dummy1 <- **as.numeric**(x[,1] > -0.5 **&** x[,1] <= 0)
dummy2 <- **as.numeric**(x[,1] > 0 **&** x[,1] <= 0.5)
 
 
# *generate environment factors*
e = **cbind** (dummy0,dummy1,dummy2)
 
# *set up the design matrix for the interaction model*
x=**cbind**(dummy0,dummy1,dummy2,x)
**for** (i in (**q**+1):(p+**q**)) {
**for** (j in 1:**q**) {
x=**cbind**(x,x[,j]*x[,i])  }
}
 
x=**scale**(x)
 
ll=0.4
ul=0.8
coef1=**runif**(**q**,ll,ul) # *for interaction effects*
coef2=**runif**(**q**,ll,ul) # *for interaction effects*
coef3=**runif**(**q**,ll,ul) # *for interaction effects*
coef4=**runif**(7,ll,ul) # *for E and G main effects*
**coef**=**c**(coef4,coef1,coef2,coef3)
**mat**=x[,**c**(1,2,3,5,7,10,15,(p+**q**+1):(p+**q**+3),
(p+5*q+1):(p+5*q+3),(p+10*q+1):(p+10*q+3))]
 
**for**(u in 1:k){
y[,u] =  0.6+rowSums(coef*mat) }
sig1 = **matrix**(0,k,k) # *AR(1) correlation*
**diag**(sig1)=1
**for** (i in 1: k)  {
**for** (j in 1: k)   { sig1[i,j] = rho^**abs**(i-j) }
}
error = mvrnorm(n,**rep**(0,k),sig1)
y = y + error
**index**=1+**c**(1,2,3,5,7,10,15,(p+**q**+1):(p+**q**+3),
(p+5*q+1):(p+5*q+3),(p+10*q+1):(p+10*q+3))
dat = **list**(y=y,e=e,g=g,**index**=**index**,**coef**=**coef**)
**return**(dat)
}

Next, we used the following sample R codes to generate a dataset with 250 subjects (*n* = 250), 75 genetic factors (*p* = 75) and 3 environment factors (*q* = 3). Moreover, there were five time points (*k* = 5). Repeated measurements were assumed to be correlated with the AR(1) structure, with a correlation coefficient ρ of 0.5. The output from the R console is given below:

> **library**(interep)
> **library**(MASS)
> **set**.seed(1000)
> n=250;p=75;k=5;**q**=3;rho=0.5
> dat=Data1(n,p,k,**q**,rho)
> e=dat**$**e
> g=dat**$**g
> y=dat**$**y
> **dim**(e)
[1] 250   3
> **dim**(g)
[1] 250  75
> **dim**(y)
[1] 250   5
> dat**$coef**
[1] 0.5934948 0.7166512 0.7787346 0.4494069 0.4576405
[6] 0.6088111 0.7519327 0.7800568 0.7447117 0.5241370
[11] 0.6688325 0.6749083 0.7816159 0.6365433 0.7102527
[16] 0.4383118
> dat**$index**
[1]   2   3   4   6   8  11  16  80  81  82  92  93  94
[14] 107 108 109
 

The R codes dat$coef provided the exact coefficients used to simulate repeated measurements in the data-generating model. These coefficients are reproducible since we set the seed before calling the function Data1. With 75 genetic factors and 3 environmental factors, the total number of main and interaction effects was 304, including the intercept. The last row of the R output shows the locations of these true effects in the vector of regression coefficients of length 304. Then, with fixed tuning parameters, we adopted the function interep to estimate the regression coefficients using the sparse interaction method with exchangeable working correlation. As the positions of true effects on the vector of regression coefficients were known and saved in the variable index.true, we were able to obtain the number of true positives (TP) and false positives (FP).

**index**.true=dat**$index**
beta0=**rep**(0.1,1+**q**+p+p*q)
lambda1=0.45
lambda2=1
**beta**.est = interep(e, g, y,beta0,corre="e",pmethod="mixed",
lam1=lambda1,lam2=lambda2,maxits=30)
**beta**.est[**abs**(**beta**.est)<0.05]=0
**index**.est = **which**(**beta**.est != 0)[-1]
tp = **length**(**intersect**(**index**.true, **index**.est))
fp = **length**(**index**.est) - tp

The TPs and FPs were printed in the R console. For the convenience of comparison, we show the locations of true and estimated non-zero regression coefficients, which are saved in variables index.true and index.est, respectively. The intercept, which corresponds to the 1st position, is not included in the two index vectors. The first 20 entries of beta.est from the R console are also provided below.

> tp
[1] 13
> fp
[1] 2
> **index**.true
[1]   2   3   4   6   8  11  16  80  81  82  92  93  94
[14] 107 108 109
> **index**.est
[1]   2   3   4   6   8   9  11  14  16  92  93  94 107
[14] 108 109
> head(**beta**.est,20)
        [,1]
[1,] 0.5962702
[2,] 0.1635084
[3,] 0.2501227
[4,] 1.2758346
[5,] 0.0000000
[6,] 0.7307669
[7,] 0.0000000
[8,] 0.6030248
[9,] 0.4935733
[10,] 0.0000000
[11,] 0.5921602
[12,] 0.0000000
[13,] 0.0000000
[14,] 0.6130440
[15,] 0.0000000
[16,] 0.6448346
[17,] 0.0000000
[18,] 0.0000000
[19,] 0.0000000
[20,] 0.0000000

Now, we briefly analyze the identification performance of the proposed method under the current replicate of data. The 2nd, 3rd and 4th positions of beta.est, as well as the true coefficient vector, correspond to the environmental main effects. By comparing index.est with index.true, we can observe that the 9th and 14th genetic effects are false positives, with regularized estimates 0.4936 and 0.6130, as indicated by beta.est. The true main effects, at positions 6, 8, 11 and 16, are all identified. The group of interactions corresponding to positions (80,81,82) is not selected. For a data-generating model with n=250,ρ=0.8,p=75 (leading to a total dimension of 304), we can record the running time with fixed tunings over 100 replicates using the following R codes:

reps=100
**time**=**rep**(0,reps)
**for** (h in 1:reps) {
n=250;p=75;k=5;**q**=3;rho=0.5
dat=Data1(n,p,k,**q**,rho)
e=dat**$**e
g=dat**$**g
y=dat**$**y
beta0=**rep**(0.1,1+**q**+p+p*q)
lambda1=0.5
lambda2=0.5
**start**_**time** <- Sys.**time**()
b = interep(e, g, y,beta0,corre="e",pmethod="mixed",lam1=lambda1,
lam2=lambda2,maxits=30)
**end**_**time** <- Sys.**time**()
**time**[h]=**as**.**numeric**(**end**_**time**)-**as**.**numeric**(**start**_**time**)
}
**mean**(**time**);**sd**(**time**)

The *interep* package has thus implemented six approaches—the simultaneous selection on both the individual and group levels under the exchangeable correlation (A1); the AR(1) correlation (A2) and independent correlation (A3); as well as A4–A6, denoting the incorporation of the exchangeable, AR(1), and independent workingcorrelation to conduct individual-level selection only. The computational time for all the methods is provided in Table 1 below.

In Zhou et al. [13], we analyzed the longitudinal lipidomics data generated by King et al. [16] using methods implemented in the *interep* package. To avoid a re-analysis of the same data leading to identical findings reported in Zhou et al. [13], we have not pursued real data analysis in this study.

## 5. Discussion

In this article, we present the R package *interep* that implements the proposed and alternative methods from Zhou et al. [13] for high-dimensional interaction analysis with repeated-measurement data. Although the regularization methods were initially motivated by a lipidomics study [16,39,40], the R package is broadly applicable when interaction effects are in the form of groups and genetic factors are of high dimensionality. We adopt MCP as the baseline penalty function for all methods implemented in package *interep*. Other baseline penalties such as LASSO, adaptive LASSO and SCAD will be considered in the future updates of the package.

We acknowledge that there exists a wide diversity of statistical methods, not necessarily within the regularization framework, that can be applied for gene–environment interaction studies. For example, recently, Liu et al. [41] developed a tree-based method to detect important gene–environment interactions when genetic factors are rare variants. Furthermore, the additive main-effect and multiplicative interaction (AMMI) model has been widely adopted in agricultural studies with multi-location variety trials [42,43]. However, Liu et al. [41] cannot accommodate longitudinal traits, and the AMMI model has rooted in ANOVA, which fails when the sample size is smaller than the number of features (i.e., the total number of main and interaction effects). In addition, statistical software such as GenStat [44] and STATISTICA [45] analyze repeated-measurement data using one-way or two-way ANOVA models which, again, require a larger sample size than the number of features. Therefore, they do not work for high-dimensional repeated-measurement data. Comparisons with methods or software that fail under high-dimensional settings are not possible. To the best of our knowledge, the most relevant alternatives have been implemented in the package *interep*.

Our biased search shows that only a very limited number of R packages are available for variable selection in high-dimensional longitudinal studies. The advantage and power of the *interep* package lies in its incorporation of group-wise sparsity in interaction studies with repeated measurements. It is also user-friendly in that the G and E factors can be specified separately as the input arguments. Other relevant packages, such as the *PGEE* package [27], focus on main effects and cannot be directly adopted for longitudinal interaction studies. Currently, all approaches implemented in the *interep* package have been developed within the generalized estimating equation (GEE) framework. The regularized GEE under interaction models can be efficiently solved using the Newton–Raphson algorithm, as demonstrated by the numerical experiments conducted in this study and in Zhou et al. [13].

The scale of longitudinal data is determined by the dimensionality of G and E factors and the sample size, as well as number of repeated measurements, which jointly affect the computational speed of package *interep*. In general, regularized variable selection works well when the number of features is reasonably larger than the sample size. Thus, a pre-screening step is necessary if the G factor is ultra-high-dimesional [46]. Environmental main effects are not subject to selection as E factors are usually determined in advance based on existing studies. As the selection is performed in the group manner, the number of E factors considered should not be excessively large or the stability of group-wise selection is sacrificed. In addition, although we set the time points (i.e., the number of repeated measurements) to five in the simulation study, the case study presented in Zhou et al. [13] shows the superior performance of the package when a phenotypic trait has been measured over 10 consecutive weeks. The trait is densely measured as the number of time points further increases. Therefore, an alternative is to consider functional data analysis [47].

The problem of missing data often occurs in longitudinal studies. There are three well-acknowledged missing mechanisms: missing completely at random (MCAR), missing at random (MAR) and missing not at random (MNAR) [48,49]. In practice, it is critical to evaluate the missing mechanisms through the sensitivity analysis before model training. Currently, the *interep* package cannot directly process repeated measurements with missing data. Users of the package have to manually remove missing observations before further analysis. We will explore incorporating missingness, especially MCAR, into the current interaction analysis in the near future.

Robustness to outliers and model misspecification is critical for the success of G × E studies. Although adopting robust loss/likelihood functions can safeguard long-tailed distributions in phenotypes [10,12], the varying coefficient models and their extended family have been systematically examined to capture G × E interactions beyond the linear assumption [50,51,52]. In repeated-measurement studies, the identification of nonlinear interactions within the GEE and QIF frameworks is also robust to misspecifications of working correlations. QIF has improved efficiency over GEE when the working correlation is misspecified. Although relevant models have also been proposed in the literature, our limited search of R packages (through CRAN) indicates that the corresponding codes are not available in the form of R packages. The release of the R package *interep* will benefit practitioners from a diversity of fields in which interaction analysis of longitudinal data is of interest.

## Figures and Tables

**Table 1:** Computational time (in seconds) under fixed tuning parameters with different combinations of sample size *n* and numbers of genetic factors *p*. The total dimension is pq+p+q+1 with *k* = 5 time points and *q* = 3 environmental factors.

		n=250		n=500	
		p=75	p=150	p=150	p=300
ρ = 0.5	A1	0.99 (0.10)	3.2 6(0.35)	4.90 (0.28)	19.05 (2.12)
	A2	1.26 (0.29)	4.85 (1.51)	7.82 (2.96)	44.35 (22.40)
	A3	0.80 (0.06)	3.01 (1.26)	4.43 (0.26)	23.11 (1.75)
	A4	0.92 (0.18)	2.84 (1.17)	4.44 (0.47)	18.05 (2.66)
	A5	1.49 (0.48)	5.54 (2.30)	13.40 (5.00)	48.31 (28.75)
	A6	0.87 (0.09)	2.83 (0.64)	4.47 (0.32)	21.61 (2.19)
ρ = 0.8	A1	0.99 (0.10)	3.27 (0.39)	4.9 2(0.39)	18.43 (2.49)
	A2	1.27 (0.38)	4.83 (1.85)	8.89 (3.57)	42.62 (21.26)
	A3	0.88 (0.44)	3.21 (1.50)	4.58 (0.26)	23.40 (1.79)
	A4	0.89 (0.16)	2.53 (0.24)	4.43 (0.39)	17.79 (3.27)
	A5	1.43 (0.54)	5.45 (2.93)	11.59 (5.55)	53.22 (29.40)
	A6	0.88 (0.09)	2.60 (0.34)	4.58 (0.34)	14.58 (8.26)

## Data Availability

The simulated data can be reproduced by rerunning the R codes presented in the article.

## Abbreviations

The following abbreviations are used in this manuscript:

GEE	Generalized estimating equation
LASSO	Least absolute shrinkage and selection operator
PGEE	Penalized generalized estimating equation
PQIF	Penalized quadratic inference function
MCP	Minimax concave penalty
SCAD	Smoothly clipped absolute deviation
SNP	Single-nucleotide polymorphisms
CNV	Copy number variations
QIF	Quadratic inference function
